# A Rent in the Left Ventricle: A Sea-Saw Between Life and Death

**DOI:** 10.7759/cureus.30665

**Published:** 2022-10-25

**Authors:** Suman Rastogi, Nava R Sharma, Bandana Rastogi, Madalasa Pokhrel, Jagdish Sharma

**Affiliations:** 1 Emergency Medicine, Sikkim Manipal Institute of Medical Sciences, Gangtok, IND; 2 Medicine, Manipal College of Medical Sciences, Pokhara, NPL; 3 Medicine, National Medical College and Teaching Hospital, Birgunj, NPL; 4 Internal Medicine, Montefiore Medical Center, New Rochelle, New Rochelle, USA

**Keywords:** stab injury, traumatic cardiac arrest, penetrating cardiac injury, cardio thoracic surgery, cardiology research

## Abstract

Penetrating injuries to the precordium are life-threatening and require early detection and immediate intervention. We present a case of penetrating cardiac injury who presented with a definitive airway and hemodynamically unstable. During the primary survey, the patient had a cardiac arrest with pulseless ventricular tachycardia. The patient was resuscitated as per advanced cardiac life support (ACLS) and advanced trauma life support (ATLS) guidelines with manual digital compression at the penetrating site leading to a return of spontaneous circulation (ROSC). After ROSC, he was shifted for emergency explorative median sternotomy. During the sternotomy, we found a clotted rent in the anterior wall of the left ventricle, which was repaired. Aggressive resuscitation and appropriate management strategy in the emergency department (ED) resulted in a successful outcome, and he was discharged after 10 days of hospital stay. Our case highlights the importance of early diagnosing and managing penetrating cardiac trauma.

## Introduction

Among all patients admitted for trauma, 10% of cases have chest trauma [1]. Penetrating injuries to the chest are primarily due to road traffic accidents involving pedestrians, but violence like shooting has higher mortality [2]. Patients with penetrating cardiac injury arrive in ED in impending or complete cardiac arrest situations with a mortality rate of almost 80% [3]. Outcomes of such patients alter significantly with an early arrival to ED, prompt diagnosis, and effective intervention [4]. We report an interesting case of penetrating cardiac injury who presented in impending cardiac arrest. He was resuscitated successfully despite having left ventricular rent with unstable hemodynamics.

## Case presentation

A 36-year-old intubated male was presented to our ED in a gasping state with a Glasgow Coma Scale (GCS) of E1VTM2. He was allegedly assaulted with a knife in the left sixth intercostal space medial to the mid-clavicular line (MCL). The airway position was confirmed in the emergency room, and suctioning was done. His saturation was 99%, with bilateral equal air entry with ventilator support. There was no evidence of bilaterally subcutaneous emphysema, bony crepitus, and lung sliding. He was pale, and his blood pressure was not recordable. Heart sounds were muffled and distant. Intravenous crystalloids and uncrossed and unmatched "O" negative blood were transfused. The patient was pulseless with a cardiac monitor showing ventricular tachycardia. High-quality Cardiopulmonary Resuscitation (CPR) was started, and the patient was cardioverted with 200 Joules. The patient bled from the penetrating site, dislodging the clot and relieving cardiac tamponade. Return of spontaneous circulation (ROSC) was achieved after two cycles of CPR, and tight occlusive compression dressing was done around the penetrating site. After ROSC, he was stable hemodynamically with a transfusion of blood products (packed RBC, fresh frozen plasma, and platelets) in the ratio of 1:1:1. His GCS was still E1VTM3. His pupil was bilaterally reactive to light, and there were no lateralizing signs.

His point-of-care ultrasound (POCUS) revealed mild pericardial effusion with normal left ventricular contraction and a thick clot covering the rent in the anterior wall. X-ray was suggestive of left lower lung contusion. Contrast-enhanced computerized tomography (CECT) thorax revealed moderate pericardial effusion with high attenuation suggestive of hemopericardium and left-sided mild hemothorax but without significant vessel injury. The patient was treated with tetanus toxoid, tetanus immunoglobulin, analgesics, and broad-spectrum antibiotics.

**Figure 1:**
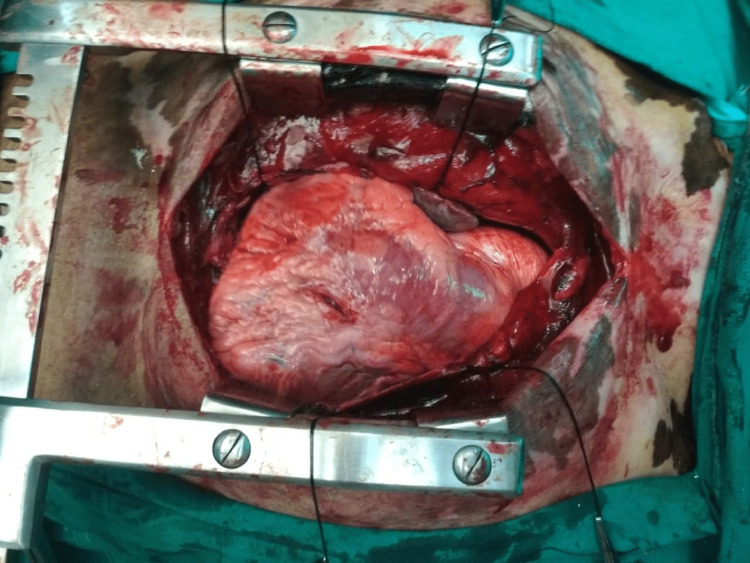
Rent in the anterior wall of the left ventricle

Focused assessment with sonography for trauma (FAST) examination and X-rays were suboptimal. Therefore, the patient was planned for exploration under general anesthesia through median sternotomy. Pericardial clots and a 0.5 cm small rent were noted (Figure 1) in the left ventricle, which was repaired successfully. The patient was admitted to the postoperative ICU, where his condition improved and he was discharged after 10 days.

## Discussion

Among penetrating injuries to the heart, the right ventricle is affected more commonly (43%) than the left ventricle (34%) due to its anterior anatomical location [5]. In 20% of cases, the left or right atrium is affected [5]. Life-threatening hemorrhage and cardiac tamponade commonly occur after penetrating cardiac injury. Pericardial defects, which communicate with the pleura, can present as hemothorax. A linear defect is common in stab injuries, which may get sealed, with intra-pericardial hemorrhage progressing to cardiac tamponade [6]. FAST examination detects cardiac tamponade. The classic finding of Beck’s triad in cardiac tamponade consists of muffled heart sounds, hypotension, and distended neck veins [6-8]. Without FAST, diagnosing cardiac tamponade is difficult because various mechanisms compensate for hemodynamic effects. Sinus tachycardia is the most commonly seen sign of pericardial tamponade, whereas hypotension is a sign of an emergent need for surgical intervention.

Pericardiocentesis is a temporary measure in cardiac tamponade secondary to penetrating cardiac injury. It is preliminary to formal thoracotomy if there are unavoidable delays to definitive surgery. ED thoracotomy is indicated in hemodynamically unstable patients who show a palpable pulse and BP, reactive pupil, purposeful movement, and an organized cardiac rhythm in the field or ED [9]. The left anterolateral approach is followed in ED thoracotomy, allowing quick access to the pericardium and heart. An incision is made in the left fourth or fifth ICS corresponding below the inframammary fold in females and nipples in males across all the intercostal musculature layers from the sternum to the posterior axillary line. After retraction and exposing the thorax, direct bleeding, laceration, or clots are examined. Penetrating cardiac injury bleeding can be controlled by direct digital pressure. A finger should never be inserted, as it may extend the wound. Continuous or interrupted sutures should suture the rent. In complex and severe lacerations, it can be temporized using a Foleys catheter and inflated, filling a large circular defect with gentle traction on the catheter. Purse string suture should be placed around the Foley’s catheter, and after removing the catheter, the suture is tightened, which seals the defect. After resuscitative ED thoracotomy, if patients are hemodynamically stable, they can be transferred to the operating room for definitive repair. Seventy percent to 80% of stab wounds and 30% to 40% of gunshot wounds survivors make it to the operating room [9,10]. Tranexamic acid 1 gm intravenous should be administered to patients with penetrating cardiac injury if presented within three hours of injury [11].

## Conclusions

Penetrating cardiac injury requires early detection and immediate aggressive resuscitation. Tight occlusive compression over penetrating wound helps seal the defect and control bleeding. Pericardiocentesis and ED thoracotomy are temporary measures performed in penetrating cardiac injury patients with impending cardiac arrest. Early identification and management of a case of cardiac tamponade are crucial for a good outcome in a patient with penetrating cardiac injury.
